# Correspondence

**Published:** 1903-04

**Authors:** Ludwig Weiss

**Affiliations:** Secretary of Committee, New York


### CORRESPONDENCE.

NEW YORK, March 4, 1903.

*Editor Atlanta Journal-Record of Medicine, Atlanta, Ga.*

DEAR SIR :—At the last (fifty-third) meeting of the American Medical Association, held at Saratoga Springs, June 10-13, 1902, a joint resolution from the Sections of Cutaneous Medicine and Surgery and Hygiene and Sanitary Science was introduced in the House of Delegates as follows :

"Whereas, There is a burning necessity to check the spread of venereal diseases, and, assuming that the States cannot with impunity ignore the condition, it lies in the province of the medical profession to discuss and recommend to the respective State Legislatures and Municipalities means not regulamentative, but social, economic, educative and sanitary in their character, to diminish the danger from venereal diseases.

Resolved, That the Section on Cutaneous Medicine and Surgery of the American Medical Association invite the Section on Hygiene and Sanitary Science to cooperate with the Section on Cutaneous Medicine and Surgery in bringing about a propaganda in the different States, looking toward a proper recognition of the dangers from venereal diseases, and to arrange for a national meeting under the auspices of the American Medical Association for the prophylaxis of venereal diseases, similar to the International Conference for the Prophylaxis of Venereal Diseases, which meets again this year at Brussels, under the authority of the Belgian Government."

This was later submitted to the House of Delegates, which endorsed the action of Section and adopted the following:

"Resolved, That a joint committee of six from the Sections on Hygiene and Sanitary Science and Cutaneous Medicine and Surgery be appointed by the President to stimulate study in and uniform knowledge of the subject of the prophylaxis of venereal diseases and to present to the American Medical Association a plan for a national meeting, similar to the International Conference for the Prophylaxis of Venereal Diseases, which meets again this year in Brussels, under the auspices of the Government of Belgium."

The committee on Prophylaxis of Venereal Diseases consists of : Dr. Henry D. Holton, Chairman, Brattleboro, Vt.; Dr. Ludwig

Weiss, Secretary, 77 East 91st St., New York; Dr. George M. Kober, 1600 T St., Washington, D. C.; Dr. W. H. Saunders, Montgomery, Ala.; Dr. L. Duncan Bulkley, 531 Madison Ave., New York City; Dr. Frank H. Montgomery, 100 State St., Chicago, Ill.

The peculiar social, racial and political conditions of our country are so different from those on the continent that they necessitate an expression of solely American ideas on this mooted question, both from a socio-economic and sanitary point of view.

The committee desires the support of the medical profession and the aid and powerful collaboration of the medical press of the country to help them in this work. It takes the liberty of soliciting expressions and views editorially and otherwise, and would be glad of personal correspondence from those supporting the movement and who will contribute by papers, etc., to make it a success in case the House of Delegates should favor the holding of such a Congress.

By giving this a place in your esteemed paper, the committee feel that you will have aided materially in forwarding the work entrusted them.

I remain with thanks, very truly yours,

LUDWIG WEISS, M.D.,
Secretary of Committee.

---

### A FORTUNATE DOG.

Raymond Hitchcock says that while he was lying in a Philadelphia hospital three or four weeks ago, convalescing from an operation for appendicitis, one of those fool friends who always say the wrong thing in the wrong place called on him and told him the following story to cheer him up: Philadelphia's most famous appendicitis expert has a dog of which he thinks a great deal, which had a lop-sided walk. A friend asked the doctor on one occasion the cause of this. "Why," was the reply, "he's got appendicitis." "Then why don't you operate on him?" queried the caller. "What! operate on that dog! Why, that dog's worth a hundred dollars."—*N. Y. Commercial.*

